# Effect of EDDY and manual dynamic activation techniques on postoperative pain in non-surgical retreatment: a randomized controlled trial

**DOI:** 10.1186/s12903-022-02702-4

**Published:** 2023-01-03

**Authors:** Selen İnce-Yusufoğlu, Neslihan Büşra Keskin, Gülşah Uslu, Dilek Helvacioglu-Yigit

**Affiliations:** 1 Department of Endodontics, Faculty of Dentistry, Ankara Yıldırım Beyazıt University, Ankara, Turkey; 2 Private practice, Idadent Oral and Dental Health Clinic, Canakkale, Turkey; 3 College of Dental Medicine, QU Health, Qatar University, P.O. Box: 2713, Doha, Qatar

**Keywords:** EDDY, Manual dynamic activation, Postoperative pain, Retreatment, Sonic activation

## Abstract

**Background:**

During non-surgical retreatment process, the products such as dentin debris, root canal fillings, irrigation solutions, microorganisms and remaining pulp tissues can extrude to the apical area and can cause the postoperative pain and flare-up. Thus, the aim of this study was to evaluatethe effect of EDDY and manual dynamic activation (MDA) techniques on postoperative pain (PP) associated with retreatment.

**Methods:**

Ninety patients scheduled for retreatment were treated at a single visit. Non-vital mandibular premolar teeth diagnosed with asymptomatic apical periodontitis were included in this study. The patients were divided into two groups (*n* = 45) on the basis of the need for additional irrigation activation procedures (EDDY and MDA). The patients’ post-treatment pain levels were asked to rate the intensity of their pain on a 10-point numerical rating scale (NRS) at the 12th, 24th, 48th, and 72nd h and 7 days.The data were analyzed using the chi-square and Wilcoxon signed-rank test was used for within-group comparisons and Mann Whitney U test was used to compare the groups by time period.

**Results:**

The difference in postoperative pain intensity between two groups was statistically significant at 12, 24, 48, and 72 h (*p* < 0.05). There was no significant difference at 7 days. Moreover, no statistically significant difference was found between the two groups in terms of gender and analgesic medication intake (*p* > 0.05). Pain intensity after the treatment was lesser in the MDA groupthanin the EDDY group at 12, 24, 48, and 72 h.

**Conclusion:**

This study could lead us to conclude that the two activation systems can be used during endodontic retreatment with no difference at PP 7 days later. However, a comparison of the groups indicated that the EDDY resulted in significantly more PP at 12, 24, 48, and 72 h.

*Trial registration* ClinicalTrials.gov Identifier: NCT04726670.

**Supplementary Information:**

The online version contains supplementary material available at 10.1186/s12903-022-02702-4.

## Background

Success in non-surgical retreatment is the removal of the contaminated previous root canal filling followed by the re-shaping, disinfection and finally hermetic filling of the root canals. Mechanical enlargement is not sufficient for widening and cleaning root canals because of the inability to make contact with all the dentin walls. Chemomechanical preparation is needed for many reasons, such as debris removal, canal disinfection, and lubricant effects during root canal treatment [1]. Nickel–titanium (Ni–Ti) systems are safe, effective, and less time-consuming for gutta-percha removal during non-surgical retreatment. However, no preparation system can completely remove gutta-percha and sealers from the canal walls [2]. Thus, ultrasonic and sonic activation systemswith various methods have been developed to more effectively remove gutta-percha and canal sealers from root canals. The goals of these systems are to strengthen canal disinfection, to increase the penetration of the solution into the apical delta and lateral canals, and to reduce the resistant bacterial strains that present difficulties for disinfecting the dentinal tubules [2, 3].

Manual dynamic activation (MDA), passive ultrasonic irrigation (PUI), and sonic irrigation (SI) are the most frequently used activation techniques [4]. In the preparation of the root canal system, gutta-percha, which has the same diameter as the final file, is a dynamic irrigation form because of the hydrodynamic activity that it creates in the root canal through oscillation in the irrigation solution [5, 6]. The MDA technique enhances debridement by improving the contact between the irrigation agent and the root canal walls [6]. EDDY is an activation technique with a sonic system that provides three-dimensional movement of the tip with the cavitation and acoustic streaming movement like that of ultrasonic devices. Irrigation solutions make contact with the dentin walls to clear the complex root canal anatomy without the limitations of ultrasound-powered devices [7].

These activation methods and preparation techniques have been found to cause inflammation in the periapical tissues. A certain amount of debris is extruded from the apical during the preparation for or removal of a root canal filling [8–10]. These methods are also associated with postoperative pain (PP) because of potential damage to the periodontal tissues [10]. Pain prevalence after endodontic procedures has been reported to be 3–58% [11]. Especially in asymptomatic patients, the occurrence of PP creates a negative situation for clinicians and patients as well. In addition, the flare-up incidence and PP in non-surgical retreatment cases is reported to be significantly higher than in primary root canal treatment [12, 13]. Selection of the instruments, preparation and activation techniques, devices, and irrigants used during treatment are important for preventing post endodontic pain. For these reasons, the aim of the study was to compare the PP intensity after using EDDY and Manuel Dynamic Activationirrigation activation techniques in mandibular premolar teeth diagnosed with previously treated andasymptomatic apical periodontitis.In the currently available literature, there is no study examining the effect of the EDDY sonic activation device on PP in non-surgical retreatment cases. The null hypothesis in this study was that there would be no difference in pain levels after usingEDDY and Manuel DynamicActivationirrigation activation techniques.

## Methods

### Sample size calculation

The sample size was based on apilot study data that indicated that 38 patients would be sufficient for each group (type I alpha error = 5%, effect size = 0.7, power = 80%). To compensate for possible dropouts during the treatment and/or follow-up periods, 45 patients were assigned to each group.

### Eligibility criteria

**Fig. 1:**
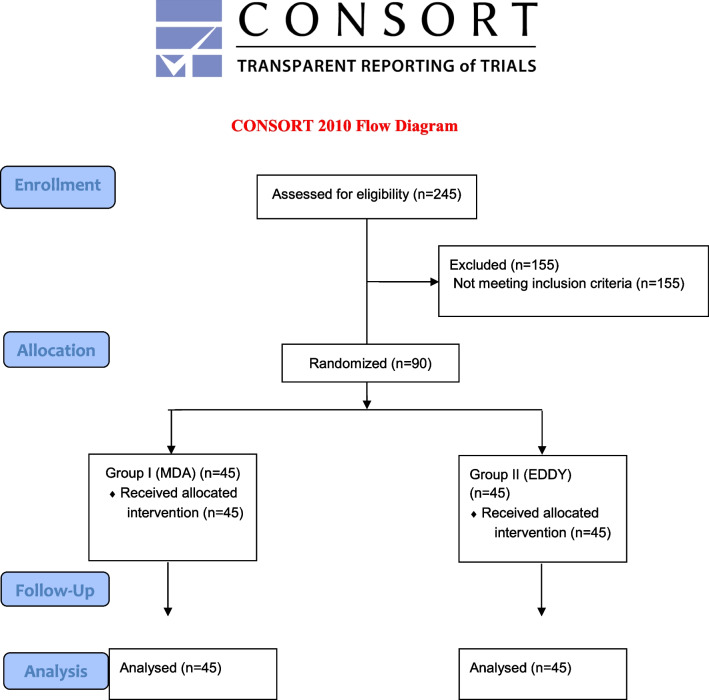
Flow diagram CONSORT for randomized clinical trials

In this study, Consolidated Standards of Reporting Trials guidelines were followed (Fig. 1) (Additional file 1). This double-blind, single center, randomized clinical trial protocol was approved by the Ankara Yıldırım Beyazıt University Ethical Board of Clinical Trials and Non-interventional Research (2019-49) and registered on www.clinicaltrials.gov (ClinicalTrials.gov Identifier: NCT04726670Registration date: 27/01/2021). Informed consent was obtained from all subjects.Patients referred to the endodontic clinic were asked to complete a questionnaire to provide pre-treatment clinical conditions and demographic data.

The sample comprised patients requiring non-surgical retreatment in previously treated mandibular premolar teeth with asymptomaticapical periodontitis.

The inclusion criteria were as follows:

1. Healthy individuals between aged 18 and 50 years.
2. Previously root canal treated mandibular premolar teeth with asymptomatic apical periodontitisconfirmed by clinical and radiolographic examination.
3. Single rooted mandibular premolar teeth with single canal that were confirmed by periapical radiographs.
4. Teeth with previous root canal obturation 2–4 mm short of the apex.
5. Periapical radiolucency (PAI 3–4) detected [14] by radiographic examination of designated teeth.
6. Presence of intact coronal restoration.
7. Teeth on which root canal treatment had been performed in the researcher’s hospital at least 4 years before the study.

The exclusion criteria were as follows:

1. Use of drugs, such as analgesics, anti-inflammatories, and antibiotics, for pain and infection control in the previous 12 h.
2. A history of susceptibility or adverse reactions to any drugs or materials used in the study.
3. Teeth with open apexes and resorption.
4. Teeth with post-core restoration.
5. Vertical root fractures and teeth on which surgery had been performed.
6. Pregnancy and breastfeeding.

### Non-surgical retreatment protocol

#### Randomization

A total of 90 patients were randomly divided into 2 groups, The randomization protocol was implemented on www.randomizer.org, a free resource for researchers to generate random numbers or randomly assign participants to experimental conditions. Patients were assigned to study groups in accordance with the number sequence obtained on the site.

A single clinician performed non-surgical retreatment on each of the 90 teeth during a single visit.A 27-gauge dental injector (Set Inject; Set Medical Instruments, Istanbul, Turkey) was used to anesthetize the patients with a solution of 40 mg Articain and 0.006 mg/mL epinephrine (Ultracain DS Forte; Aventis, Istanbul, Turkey). Old restorations and caries were removed, and on the basis of the straight-line principle, round diamond burs were used to create access cavities under rubber dam isolation. All subsequent treatment procedures were performed under rubber dam isolation, and 3.5× (ZumaxSle Loupe) magnification. When necessary, the cervical margin was elevated with composite resin to ensure the continuity of the isolation.

The gutta-percha and sealer was removed with hand files and ProTaper retreatment (DentsplyMaillefer) files (D1-D3) with an endodontic motor (Sybronendo,Gulsa, Turkey), at 550 rpm and 2.5 Ncm torque for mechanical preparation. No solvent was used.The working length was determined with using a #15 K file (Dentsply, Sirona, Ballaigues, Switzerland) and the ProPexPixi apex locater (DentsplyMaillefer). Periapical radiography confirmed the working length the removal of the canal filling. Root canals were enlarged to the ProTaper Universal F3 (30/0.9) size with an endodontic motor (Sybronendo,Gülsa, Turkey), at 300 rpm and 2 Ncm torque. A lubricant (Glyde File Prep, DentsplyDeTrey, GmbH, Konstanz, Germany) was used between each file to prevent the rotary files from getting stuck in the root canal. A total of 20 mL 2.5% sodium hypochlorite (NaOCl) irrigation solution (Werax, Izmir, Turkey) was used for each treatment and irrigation was delivered performed withusing an open-ended 27- gauge irrigation needle. For all teeth, the final irrigation was performed with 5 mL 17% ethylenediaminetetraacetic acid (EDTA)solution (Werax, Izmir, Turkey) for 1 min. After the non-surgical retreatment procedure, the patients were divided into two groups on the basis of the additional irrigation activation procedures with 6 mL 2.5% NaOCl (*n* = 45) (Fig. 1). Information on which irrigation activation methods to use was not given to the patient.

*First group *(*manual dynamic activation-MDA*) A final rinse with 6 mL 2.5% NaOCl was performed after shaping, using a ProTaper Universal F3 gutta-percha (Dentsply, Maillefer, Ballaigues, Switzerland) with up and down movements and a 2 mm amplitude at a frequency 100 strokes to as 1 mm short from the working length for 1 min.

*Second group *(*EDDY*) A 28 mm long polyamide tip with 25.04 size and taper was adapted to TA-200 (Micron, Tokyo, Japan) and operated at 6,000 Hz and, the maximum speed setting [15] and it was placed in the canal 2 mm shorter from the working length. 6 mL 2.5% NaOCl was administered to the canal following three 20-second activation–nonactivation cycles (2 ml/20 sec).

The root canals were dried with paper points (Dentsply, Sirona). The working length was reached usingProTaper Universal F3 gutta-percha (Dentsply, Sirona) with a tuck-back effect.The gutta-percha was then covered with sealer (AH Plus; Dentsply, Sirona), and root canal fillings were completedusing the single-cone technique.After the completion of the root canal filling procedures, the residual materials were removed with a heat source. A resin composite (3 M, ESPE) was used for the coronal restorations.Occlusal reduction was performed on all the teeth included in the study.No antibiotics or analgesics were prescribed.

### Patient questionnaire

The postoperative follow-up and evaluation of the cases were performed by a researcher who had no knowledge of the study groups.A 10-point numerical rating scale (NRS) was introduced to the patients, and they were asked to rate their post-treatment pain by telephone after 12, 24, 48, and 72 h.The antibiotics and analgesics usage were questioned.The patients were appointed for the clinical examination 1 week later.Palpation and percussion sensitivity in the treated teeth was determined on the basis of the patients’ perceptions of pain. All percussion tests were performed by the same operator to ensure standardization.The measurement values were based on the 10-point NRS. The pain scores were placed into the following four categories: 0 = none, 1–3 = mild, 4–6 = moderate, and 7–10 = severe.

### Statistical analysis

IBM SPSS Statistics for Windows, Version 21 (IBM Corp., Armonk, NY, USA) was used in the statistical analysis. The categorical variables were analyzed using Pearson’s chi-square test.According to the number of subjects in the eyes, chi-square analysis with Fisher’s exact test and Monte Carlo simulations were used. The Wilcoxon signed-rank test was used for within-group comparisons for the different time periods.To compare between groups, the normality of the data was determined using the Kolmogorov-Smirnov test, and Mann Whitney U tests were performed for comparisons. The significance level for all tests was (*p* < 0.05).

## Results

The ages of the patients ranged from 20 to 50; the mean age was 35.92 ± 13.81. Fifty-three (58%) of the 90 patients included in the study were women; 37 (42%) were male. An examination of the distribution of PP on the basis of gender indicated that there was no statistically significant difference between the groups (*p* > 0.05) (Table 1).

**Table 1:** Distribution of gender, age, and analgesic use

Variables	MDA (n = 45)	EDDY (n = 45)	p-value
*Gender (%)*			
Male	17	20	*p* > 0.05
Female	28	25	*p* > 0.05
*Age (years)*			
Mean SD	35.9 ± 13.17	35.8 ± 14.46	
Range	20–69	20–69	
Analgecis	13	20	0.095

The distribution of PP between the groups and within the groups by time period is summarized in Table 2. The incidence of moderate PP at 12 h was significantly higher in the second (38%) group than in thefirst group (*p* = 0.02). The incidence rate of “mild pain” at the 24th h was the highest: 38% in the second group. The incidence of “no pain” in the first group was 93% (*p* = 0.0001). The incidence of “mild pain” at 48 h was 29% in the second group (*p* = 0.004). At 72 h, the incidence of “mild pain” was 22% in the second group. Mild pain was not reported in the first group (*p* = 0.001).There was no significant difference in the PP values regarding day 7.(*p* > 0.05). According to the Mann-Whitney U test the second group was found statistically significant than first group all time period (*p* < 0.05, Table 2). The evaluation of PP within the groups in terms of time period is presented in Table 3. The results indicate that there was no statistically significant difference between the groups regarding post-treatment analgesic use (*p* > 0.05).

**Table 2:** A Comparison of PainLevelsat different time intervals according to the Irrigation Activation Protocols

PainLevels	MDA (n/%)	EDDY (n/%)	*p* Value
*12 Hours*			
None	26 (58%)	10 (22%)	0.002^*^
Mild	11 (24%)	14 (31%)	
Moderate	8 (18%)	17 (38%)	
Severe	0 (0%)	4 (9%)	
*24 Hours*			
None	42 (93%)	23 (51%)	0.0001^*^
Mild	3 (7%)	17 (38%)	
Moderate	0 (0%)	4 (9%)	
Severe	0 (0%)	1 (2%)	
*48 Hours*			
None	43 (96%)	31 (69%)	0.004^*^
Mild	2 (4%)	13 (29%)	
Moderate	0 (0%)	0 (0%)	
Severe	0 (0%)	1 (2%)	
*72 Hours*			
None	45 (100%)	35 (78%)	0.001^*^
Mild	0 (0%)	10 (22%)	
Moderate	0 (0%)	0 (0%)	
Severe	0 (0%)	0 (0%)	
*1 Week*			
None	45 (100%)	40 (44.4%)	0.056
Mild	0 (0%)	5 (5.6%)	
Moderate	0 (0%)	0 (0%)	
Severe	0 (0%)	0 (0%)	

*p < 0.05

**Table 3:** The evaluation of post operative pain (MeanRank) within the group according to time periods

	12 Hours	24 Hours	48 Hours	72 Hours	1 Week
MDA	3,83^a^	2,88^bcde^	2,82^bcde^	2,73^de^	2,73^e^
EDDY	4,49^a*^	3,21^b*^	2,66^c*^	2,42^d*^	2,22^e*^
*p*	0,0001	0,0001	0,001	0,001	0,022

Different superscripts indicate statistically significant difference at 5% significance level (^a, b, c , d, e^for rows)

*Statictical significance within a column (Mann–Whitney U test)

## Discussion

Multiple factors may cause post-endodontic pain such as age, gender, tooth type, presence of periapical radiolucency, presence of preoperative pain and occlusal reduction [16, 17] Intratreatment factors such as mechanical irritation due to excessive instrumentation, extrusion of infected debris and exudate into the periapical tissues can also cause PP [18]. The generation of PP due to the activation systems has been previously well studied [19, 20], however there is no study evaluated PP using EDDY during retreatment procedure. EDDY is a device that works with the newly released sonic system; thus, relevant clinical data is missing related to its effectiveness. This study aimed to compare the effects of EDDY and MDA on PP in a clinical set-up. There was no difference in PP within the first week after non-surgical retreatment using these activation systems. However, a comparison of the groups indicated that EDDY resulted in significantly more postoperative pain at 12, 24, 48, and 72 h. Thus the null hypothesis were partially rejected.

The double-blind technique was applied in the present study. The patients and evaluating clinicians were blinded about the administered treatment procedure. In addition, the statistician did not have any information about the group characteristics during the analysis. The groups were identified by assigned numbers. It has been reported that dental configurations, analgesics, and pre-operative pain are the factors related with occurrence of incidence of PP. Thus, to facilitate standardization and objectivity, the patients who has mandibular first premolar teeth treatment has been included in the present study. In addition, they had no pain and did not use analgesics before treatment. Retreatment was performed at a single visit because of the possibility of leakage of the temporary filling materials [21] and the possible effect of the interappointment duration on PP. ProTaper retreatment and ProTaper Universal F3 systems operated with a crown-down [22] system were chosen to minimize the occurrence of apical extrusions during instrumentation.

NaOCl is a widely used irrigation solution because of its antimicrobial and tissue-dissolving effects. It can be used in concentrations of 1 to 5.25%. High concentrations might increase the toxic effects on periradicular tissues [23]

and cause PP. Therefore, in the present study, a 2.5% NaOCl concentration was preferred to obtain the optimum benefits from its antimicrobial effects while minimizing its toxic and PP-inducing effects. The standardization of study protocols can mitigate the effects of intraoperative variables on outcomes.

Periapical radiolucency has been found to cause PP lasting two or more days [16]. The reason is that 83.2% of teeth with periapical lesions have foraminal resorption [24]. Thus, the disruption of apical constriction can be caused by the extrusion of irrigation solution to the periapical tissues [25]. The extrusion of irrigation solutions from the apical is associated with a burning sensation, damaged periapical tissues, and pain [9, 10]. Other studies have reported on the occurrence of PP within the first 2 days of root canal treatment and the decrease over time [19, 26–29].

A meta-analysis study investigated the PP incidence after instrumentation with rotary and reciprocating root canal file systems andstated that the rotary instruments are associated with a lower incidence of PP than reciprocating instruments [30]. Regarding that statement the rotary instruments were chosen for root canal preparation in the present study.

In both groups in our study, pain was the most intense at the 12th h. It then gradually decreased. A comparison of the groups indicated that EDDY resulted in significantly more postoperative pain at 12, 24, 48, and 72 h. There was no significant difference between the two groups during the first week. Therefore, the null hypothesis was partially rejected. Considering the evaluation within the group, the PP levels in the first group were significantly higher at the 12th h and minimal after the 24th h. In the second group, the pain level further decreased after the 48th h. In the present study, a score of 7 to 10 was considered to represent severe pain, which was characterized as a flare-up [19, 31]. It was not seen in the first group at the 12th h. However, 8.8% of the second group had severe pain, and one patient was reported to have facial swelling. The incidence of flare-ups after endodontic treatment has been found to be 1.4 to 16% [18, 32]. This indicates that atraumatic treatment protocols were realized in the present study. Gundogar et al. [33] evaluated the effects of EDDY, EndoActivator (EA) and PUI on PP in premolar teeth with irreversible pulpitis. They found no significant difference between the groups after 24 h. Erkan et al. [34] evaluated the effects of EDDY, MDA, SWEEPS and PUI on PP in root canals of premolar teeth with the diagnosis of irreversible pulpitis. They found no significant difference between the PUI, EDDY and MDA groups after 8 and 48 h but on day 7 they recorded the highest score and pain prevalencein EDDY group. On day 7 first and second groups showed similar results regarding the pain prevalence. Those findings may be attributed to the other factors related to the endodontic treatments in two studies. In the current study according to the statistical analysis of between two groups pain level was higher in second group at 12, 24, 48, and 72 h. In a previous study which evaluated debris extrusion with irrigation activation systems; EDDY, PUI and PIPS, they found that EDDY caused statistically significant debris extrusion from the apical root canals [35]. The results of the current study supported the findings of this study. The pain levelsfound higher in the second group might had been due to thedebris extrusion from the apical foramen.

A review of the literature yielded two in vitro studies on the creation of apical extrusion upon usingEDDY. EDDY was found to cause significantly more apical extrusion than ultrasonic, mechanical, and positive pressure agitation techniques [35, 36]. Aydınet et al. [37] reported that EDDY resulted in more bacterial extrusion, and there was no difference between EDDY, Standard Needle İrrigation (SNI) and EA in terms of bacterial extrusion. The efficacy of irrigation agents is enhanced by increasing the flow rate during dispersal into the complex root canal system in sonic activations [4]. It is possible that a flexible tip and high speed (5,000–6,000 Hz) might lead to apical extrusion. The three-dimensional movement results in higher flow rates, allowing the irrigation agents to advance along the root canal. A review of studies in which EA sonic systems were used indicated less apical extrusion [38] and PP [20] than were observed with conventional endodontic syringes.

In the current study, postoperative pain severity was evaluatedafter 12, 24, 48, 72 h sand 7 days with a composite measure of patient-reported and clinician-reported outcomes, that included pain scale and clinical examination in accordance with the recent clinical guidelines [39]. Pain was categorized as no pain, mild, moderate and severe using NRS [40]. The NRS is regarded as one of the best single-item methods available to quantify the intensity of pain, despite the visual analog scale (VAS) being one of the most extensively used tools to survey the severity and relief of pain [41, 42]. Because it makes it easier for patients to explain their pain, the NRS was the preffered approach of pain analysis [43, 44].

The effects of gender on PP are controversial. Some studies have reported more incidence of PP in women[16, 45]. Others have found no association between gender and PP [28, 46, 47]. In the present study, gender did not affect PP. Post-treatment analgesics were not prescribed. Ibuprofen was prescribed for patients who reported experiencing pain and requested relief when contacted by phone.There was no significant difference between the groups regarding the post-treatment analgesic intake. However, the second group was 44.4%, and thefirst group was 28.9%. The present study’s findings support those of previous studies [20, 28, 29].

The factors in pain incidence and severity remain unclear. Studies in which pain is assessed have limitations. Thus, the present study aimed to objectively evaluate a subjective experience that might be influenced by individual variability. A limitation of the present study is that a sham was not administered after the treatment to prevent the initial levels of PP from taking analgesic.

The value of our work lies in the designand validity of the methodology. Randomized controlled trials represent most scientifically rigorous method of hypothesis testing in evidence-based medicine. Despite that the findings cannot be generalized to allretreatment cases, the findings extend our knowledge and add to a growing body of literature onPP after the use of EDDY and MDA activation techniques in non-surgical retreatment. Future investigations designed as split-mouth studies,are suggested, to eliminate individual variabilities.

## Conclusion

Pain intensity and frequency after a single non-surgical retreatment session were lesser in the MDA group than the EDDY group in the asymptomatic mandibular premolar teeth. There was no difference in PP within the first week after non-surgical retreatment using these activation systems.

## Supplementary Information

**Additional file 1:** CONSORT 2010 checklist.

## Data Availability

The data in this study are available upon request from the corresponding author.

## Abbreviations

Ni**–**Ti: Nickel–titanium
MDA: Manual dynamic activation
PUI: Passive ultrasonic irrigation
SI: Sonic irrigation
PP: Postoperative pain
NRS: Numerical rating scale
EA: EndoActivator
SNI: Standard needle i̇rrigation
